# Analysis of Spinal Pilocytic Astrocytoma in 12 Case Reports and Literature Review

**DOI:** 10.5334/jbsr.3328

**Published:** 2024-09-18

**Authors:** Lin-Lin Hu, Shuang Liang, Peng Zhong, Yi Mao

**Affiliations:** 1 Department of Radiology, Daping Hospital, Army Medical University, Chongqing, 400042, China; 2 Chongqing Clinical Research Centre of Imaging and Nuclear Medicine, Chongqing, 400042, China; 3 Department of Radiology, The Second Affiliated to Mudanjiang Medical University, Heilongjiang Province 157000, China; 4 Department of Radiology, Third Affiliated Hospital of Harbin Medical University, Heilongjiang Province 150000, China; 5 Department of Pathology, Daping Hospital, Army Medical University, Chongqing, 400042, China

**Keywords:** pilocytic astrocytoma, spinal cord, magnetic resonance imaging, misdiagnosis

## Abstract

Spinal pilocytic astrocytoma (PA) is a rare disorder with atypical, clinical and imaging characteristics, and generally limited to case reports. We analysed the clinical manifestations, imaging findings, treatment and prognostic follow-up of 12 patients with spinal PA admitted from January 2010 to July 2021, and reviewed the relevant literature. Radiological assessment, especially magnetic resonance imaging, can help to provide effective diagnostic information. The diagnosis and differentiation of this disease is discussed in an attempt to contribute to a more comprehensive preoperative assessment.

## 1. Introduction

Pilocytic astrocytoma (PA) is classified as grade I astrocytoma in the 2021 WHO Classification of central nervous system tumors due to its benign biological behavior, limited growth and slow progression [1]. PA constitutes around 5% of CNS gliomas and predominantly affects children and adolescents aged 10–20 years, with no significant sex difference in incidence. The common onset site of PA is in the cerebellum and periventricle; however, it can also occur elsewhere in the intracranial midline [2–4]. Spinal cord PA cases are relatively rare, and they have an insidious growth pattern with clinical manifestations lacking specificity, leading to a high rate of misdiagnosis [5]. The clinical signs of spinal cord PA depend on the involved spinal cord segments and tumor size, with symptoms such as pain, decreased motility, numbness and decreased perception, which may overlap with other intramedullary space-occupying lesions. Given the low incidence of spinal PA, limited clinical studies with large samples, and the lack of specific clinical manifestations, confirming the diagnosis by imaging alone is challenging. Consequently, clinicians often lack a comprehensive understanding of the tumor and its treatment. To enhance the understanding of the tumor, its diagnosis and treatment, we analyzed 12 cases of spinal cord PA from our hospital to improve the understanding of the disease, its diagnosis and treatment.

## 2. Materials and Methods

### 2.1 Study selection

We collected medical records of patients diagnosed with PA of the spinal cord admitted to our hospital from January 2010 to July 2021. The data included information from electronic medical record system, imaging, and telephone follow-up. We excluded patients with a history of other tumors, patients with incomplete MRI, and patients lost to follow-up. Ultimately, 12 patients were included as the study subjects, comprising 7 males and 5 females, aged 24–66 years, and a mean age of 55.3±6.9 years. A comprehensive summary analysis was performed on these 12 patients, focusing on the preoperative clinical manifestations, imaging findings, pathological characteristics, differential diagnosis, surgical methods, and prognosis follow-up.

### 2.2 Data analyses

All imaging data were analyzed retrospectively by two associate chief physicians with over 10 years of clinical experience on a PACS workstation. In cases where their interpretations differed, a final result was reached by consensus.

### 2.3 Collected data

We collected demographic and clinical data, including patient sex, age, main complaint, treatment method, pathological findings, presence of recurrence and metastasis, and continuous tumor-free survival.

Regarding the radiological results, we evaluated and recorded the following imaging findings: (1) The site of the tumor, defined as the involved spinal cord segment, (2) Tumor growth pattern, categorized as central and eccentric, (3) The mode of tumor infiltration, categorized as limited and diffuse, (4) Maximum diameter of the tumor, (5) Signal characteristics of the lesions with different sequences on MRI plain scans, (6) Enhancement features of MRI enhanced lesions, according to the homogeneity of the tumor enhancement, it was classified as uniform enhancement and partial enhancement, and according to the degree and mode of enhancement, it was defined as no enhancement, nodular enhancement, diffuse uniform enhancement and diffuse uneven enhancement, (7) Presence of syringomyelia, defined as fluid accumulation inside and outside the ependyma of the central spinal canal, appearing in tubular series with dilatation of the central spinal canal, (8) Determination of the presence of spinal cord edema, characterized by an equal or slightly low signal with flakes in the spinal cord on T1WI and a high signal on T2WI, (9) Identification of cystic lesions and hemorrhage within the tumor. Cystic lesions were defined as areas within the tumor without enhancement, while hemorrhage was defined as a high signal on T1WI and a low signal on T2WI within the tumor (Table 1).

**Table 1:** Summary of clinical and follow-up data of spinal cord PA cases

CASE NUMBER	SEX	AGE (YEARS)	BIG OR SMALL	POSITION	SYMPTOMS	PODX	MRI EXPRESSION			SURGICAL METHOD	PATHOLOGICAL FEATURES				FOLLOW-UP TIME (MONTHS)
							T1WI FLAT SWEEP	T2WI FLAT SWEEP	ENHANCEMENT SCANNING		GFAP	EMA	PR	KI-67	
Case 1	Male	59	2.1 × 1.0 × 1.1	T10	Low back pain, Lower limb weakness and numbness for more than 10 days	Neurogenic tumor	Low signal	A slightly higher signal	Border	GTR	+	–	–	<2%	12
Case 2	Female	59	3 × 1 × 1	T6-7	Repeated low back pain for 13 years, with aggravation Hypesthesia in the left lower limb for 4 years	Ependymocytoma	High signal	High signal	Mild	GTR	+	–	–	<1%	3
Case 3	Male	45	1.0 × 1.1 × 1.0	C1	Right shoulder and neck pain with numbness of the right thumb for 1 month	Angioma	Low signal	A slightly higher signal	Tubercle	GTR	+	–	–	<1%	3
Case 4	Male	66	0.6 × 0.8 × 2.9	C3-5	Progressive weakness of the right limb, Left limb numbness for more than 40 days	Myelitis	Isosignal	High signal	Tubercle	STR	+	–	–	<1%	3
Case 5	Male	66	1.0 × 1.2 × 3.1	Conus medullaris	Progressive weakness of both lower extremities for 4 months	Ependymocytoma	Low signal	A slightly higher signal	Tubercle	GTR	+	–	–	<1%	3
Case 6	Female	43	1.3 × 1.4 × 3.4	Conus medullaris	Double lower limb weakness for more than 20 years, constipation, urine weakness for more than 5 years	Ependymocytoma	Clutter	High signal	Mild	GTR	+	–	–	<2%	22
Case 7	Female	24	9.7 ×	C1-7	Neck pain for 6 days, limb numbness and mobility disorders for 7 hours	Ependymocytoma	Clutter	Mixed high signal	Mild	STR	+	–	–	<2%	24
Case 8	Male	62	1.5 × 1.5 × 4.5	Conus medullaris	Right lower limb numbness and pain for half a year, Aggravated with the left foot numbness and half a month	Astrocytoma	Isosignal	Mixed high signal	Tubercle	STR	+	–	–	<2%	8
Case 9	Female	52	0.6 × 0.7 × 0.8	Cauda equina	Low back pain with double lower limb pain for 3 months	Molluscum simplex	High signal	Mixed high signal	Mild	GTR	+	–	–	<1%	10
Case 10	Male	29	1.3 × 1.2 × 1.1	Cauda equina	Low back pain, Lower limb weakness and numbness for more than 10 days	Glioma peripheral	Low signal	Mixed high signal	Tubercle	GTR	+	–	–	<1%	48
Case 11	Female	57	1.3 × 1.5 × 5.0	Conus medullaris	Repeated low back pain for 13 years, with aggravation Hypesthesia in the left lower limb for 4 years	Astrocytoma	Low signal	High signal	Tubercle	STR	+	–	–	<2%	14
Case 12	Male	38	1.8 × 1.2 × 0.8	Conus medullaris	Right shoulder and neck pain with numbness of the right thumb for 1 month	Astrocytoma	Low signal	High signal	Tubercle	STR	+	–	–	<2%	3

* GTR = gross total resection; STR = subtotal resection.

### 2.4 Histopathological classification

All surgical specimens were fixed with 10% neutral buffered formalin and stained with hematoxylin-eosin. The histopathological diagnosis was confirmed by pathologists with more than five years of clinical pathology experience according to the fifth edition of the WHO Classification of central nervous system tumors [6].

## 3. Results

### 3.1 Epidemiology

Twelve patients with spinal PA who met the pathological diagnostic criteria were reviewed in detail, including 7 males and 5 females, with a male-to-female ratio of 1.4:1. Patients’ ages ranged 24–66 years, with a mean age of 55.3±6.9 years and a median age of 54.5 years.

### 3.2 Clinical features

Among the 12 patients with spinal PA, 6 had limb numbness, and 4 had limb movement disorder. The most common initial clinical symptoms were limb pain, limb weakness and paresthesia (9 cases). The pain site was mainly in the lower back. One patient (Case ) had an acute onset that rapidly progressed to acute quadriplegia within a few hours, leading to a misdiagnosis of acute myelitis. Only one case had multiple lesions, while the rest had single lesions. Tumor diameters ranged 0.8–8.8 cm, with an average of 3.2 cm. All 12 patients underwent preoperative laboratory investigations, and no significant abnormalities were reported. Before surgery, 3 patients were diagnosed with neurogenic tumors, 4 with ependyma, and 1 with hemangioma. Tumors locations included the conus medullaris (5 cases), cervical segment (3 cases), thoracic segment (2 cases), and cauda equina (2 cases). Table 1 summarizes the clinical characteristics of 12 study subjects.

### 3.3 Radiological findings

**Figure 1:**
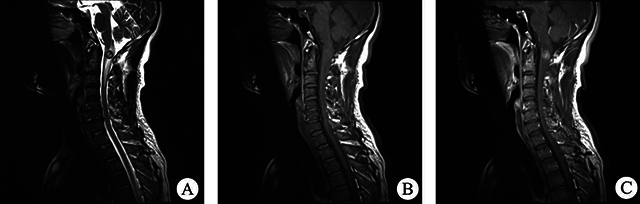
**(A)** On sagittal T2WI, the mass was located at the odontoid level and showed a mixed high-signal shadow of circular nodules with obvious strip edema around the tumor. Sagittal T1WI **(B)** and enhanced T1Wl **(C)** revealed significant enhancement of the nodular tumor on enhanced scans.

**Figure 2:**
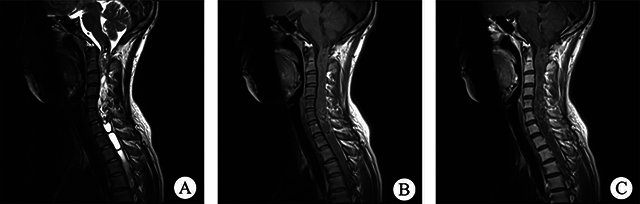
**(A)** Sagittal T2WI in the cervicothoracic segment showed a cystic spinal cavity in the T1-4 segment, irregular tumor tissue in the C1-7 layer with mixed high-signal imaging, and a rare “bleeding cap sign.” **(B)** Sagittal TIWI showed total involvement of the cervicothoracic pulp. **(C)** Sagittal TIWI enhanced scan showed mild uneven enhancement of tumor tissue, with an internally bleeding cystic lesion and no enhancement in the syringomyelic area.

**Figure 3:**
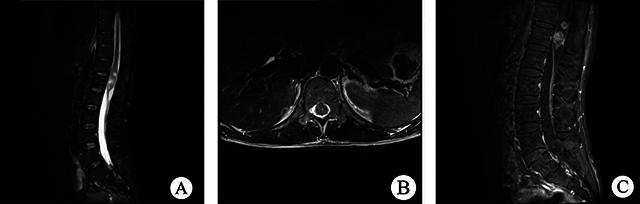
**(A)** Sagittal T2WI lesions in the lumbar segment in the conus medullaris showed mixed high-signal shadows; **(B)** Axial T2WI showed lesion involvement across the entire spinal cord. **(C)** Sagittal T1WI lipid-pressure imaging showed significant nodular enhancement.

**Figure 4:**
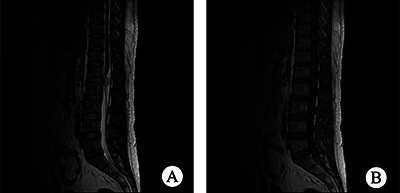
**(A)** Sagittal T2WI of the lumbar spine shows a nodular and slightly high signal shadow at the L1 level. **(B)** Sagittal TIWI enhancement showed significant lesion enhancement, closely related to cauda equina.

**Figure 5:**
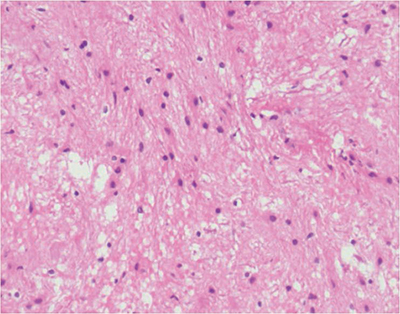
HE staining showed that scattered tumor cells can be seen in the glial background, the tumor cells are oval/short fusiform, the cytoplasm is slender at both ends, and the Rosenthal fibers can be seen in the local background.

All 12 patients had complete imaging results. These lesions were classified into two types based on their spatial relationship with the spinal cord: 10 were intramedullary, and 2 were extramedullary. Among the intramedullary lesions, 4 were categorized as intramedullary nodule type (Figure 1), and 6 as intramedullary diffuse type, based on the extent of spinal cord involvement on axial T2WI images. The intramedullary nodule-type cases were observed in the thoracic (2 cases) and cervical (2 cases) segments, showing clear boundaries with visible compressed normal spinal cord tissue. Three of them showed eccentric growth. Spinal edema was observed in all nodular lesions, with no syringomyelia, bleeding, or cystic change. Regarding intramedullary diffuse type cases (Figures 2 and 3), 5 were located in the conus medullaris and 1 in the cervical segment, involving almost all spinal sections and causing spinal cord thickening. Among these cases, 2 showed syringomyelia, 2 had spinal edema, and 2 exhibited hemorrhage and cystic changes. Two patients had extramedullary lesions (Figure 4) near the cauda equina region, presenting as nearly circular nodular lesions measuring less than 1 cm. Surgical operations confirmed that the lesions were in the spinal dura mater, with an intact lesion envelope attached to part of the cauda equina. On MRI plain scans, 12 cases showed high or mixed high signal on T2WI, 6 showed equal low signal on T1WI, and 2 showed high signal on T1WI. The enhanced scan showed edge enhancement scan in 1 case, mild enhancement in 4, and nodular enhancement in 7. None of the 12 patients showed evidence of cerebrospinal fluid spread before the operation.

### 3.4 Operative method

Posterior laminectomy of tumor tissue was performed. In this group, no individual had spinal cord, nerve root, vertebral artery, phrenic nerve, and other important structural damage due to surgery. All patients received surgical treatment; 7 underwent complete tumor resection, 5 underwent partial tumor resection, and no death was recorded.

### 3.5 Pathology

Among the 12 patients, 11 were isolated cases. The maximum tumor diameter was 1.0‒3.2 cm, and the average tumor diameter was 2.3 cm. There was no specific expression of PA on immunohistochemistry. GFAP, Vimentin and s-100 were positively expressed in 12 patients, and the Ki-67 proliferative index of all 12 patients was <5% (Figure 5).

### 3.6 Follow-up and prognosis

Postoperative routine follow-up and MRI review were performed on 12 patients to evaluate tumor resection status, recurrence, and metastasis. The follow-up time was 3‒48 months, with an average of 12.7 months. In most patients, the symptoms caused by tumor compression disappeared after treatment. However, two patients experienced lingering numbness that did not completely resolve at 6 months follow-up after discharge, likely due to the long course of the disease. Case was initially misdiagnosed as myelitis during the first visit. After initiating hormone shock, the patient’s symptoms progressively worsened, with the numbness progressing from below the lower extremities to the groin area. However, after the operation, the patient received routine symptomatic treatment, such as hormone therapy to reduce spinal edema, anti-inflammatory medication, and neuronutrition. During the follow-up period, no significant clinical signs were observed. Moreover, none of the 12 patients had local tumor recurrence, distant metastasis, or death without adjuvant treatment.

## 4. Discussion

WHO classifies PA as grade I, mainly seen in children but rarely in adults. While PA can occur in all nerve axes. However, its occurrence in the spinal cord is rare. According to previous literature reports, PA derived from the spinal cord accounts for only 9% of all adult Pas [7–9]. According to data from the Central Brain Tumor Registry of the United States (CBTRUS), PA accounts for 12.4% of all spinal cord tumors in children and adolescents. In contrast, it accounts for only 0.8% of adults (20 years and older) [10]. In this study, 12 cases of spinal cord PA were pathologically diagnosed, all of which were sporadic. Among these patients, 7 were male, and 5 were female, with ages ranging 24–66 years, and an average age of 55.3 ± 6.9 years, which was older than that reported in the literature [11, 12].This may be attributed to the relatively small number of cases included in our study.

Though rare, spinal PA has received substantial attention due to the significant neurological impairment it causes. PA first presents as movement disorders, including limbs numbness and weakness, as well as neck, shoulder, and lower back pain. The intramedullary location of spinal PA often leads to symptoms specific to its location compared to other nerve axis PAs. For instance, neck tumors may cause numbness of the upper limbs and neck and shoulder pain. Chest tumors typically lead to numbness of the lower limbs and back pain, which can progressively worsen and affect bladder and bowel function, and in severe cases, may lead to difficulty in walking. Among the 12 patients, the first symptoms were limb pain, limb weakness, and varying degrees of numbness. Due to the hidden growth of spinal cord PA, its progression can last several months to years. For example, Case in this study had a disease course spanning over 20 years. Some patients experienced a rapid onset time with severe symptoms, leading to a misdiagnosis of acute spinal cord inflammation. Case was initially misdiagnosed with acute myelitis and treated with hormone shock therapy, but symptoms persisted after a week, leading to the consideration of neoplastic lesions. The lack of specific clinical manifestations, the rarity of the condition, and its likelihood of misdiagnosis often confused with ependymoma, neurogenic tumor, or other diseases, impacting the treatment strategy. In this study, only 3 cases were considered as possible spinal cord PA before surgery, emphasizing the importance of adequate imaging diagnosis and differentiation.

MRI is the primary imaging method for diagnosing PA, as it provides relatively accurate information about the location, number, size, scope and relationship with neighboring tissues. Further, MRI provides comprehensive information for surgical decision-making and has become the most effective means for the preoperative evaluation of PA. Previous literature has shown that the cervicothoracic segment is the commonest site of spinal cord PA [13]. However, in this study, 5 cases occurred in the conus medullaris, 3 in the cervical spinal cord, 2 in the thoracic segment, and 2 in the extramedullary segment, differing from previous reports [14, 15]. Nodular PA is usually small, does not involve the whole spinal cord at the transverse position, and has clear boundaries. The reason for this appearance is that spinal PA rarely invades surrounding tissue when compared to other invasive spinal astrocytomas. Instead, it mainly exerts pressure on the surrounding normal tissues [16].Spinal PA usually grows eccentrically and protrudes outward from the surface of the spinal cord because the lesion originates from the spinal cord rather than the ependymoma cells in the central duct. In this study, 3 cases showed eccentric growth, and 1 was located in the central spinal cord. Usually, the spinal cord PA shows a low TIWI signal and a high T2WI signal. Additionally, most spinal PA lesions are accompanied by different degrees of spinal edema. Fortunately, the incidence of syringomyelia is relatively low and tends to be localized, even in cases with central duct compression or tumors in the central region. In this group of cases, the magnetic resonance plain scan signals, the presence of spinal edema, and the probability of syringomyelia were consistent with previous literature reports [17, 18]. Notably, Case exhibited small lesions with significant edema.

Diffuse lesions tend to be larger and involve the entire transverse position of the spinal cord, leading to thickening of the spinal cord. In larger tumors, the lesion signals may become mixed. She et al [19]. reported that out of 13 cases of spinal cord PA, 8 (61.5%) showed cystic changes, and the cyst wall may or may not exhibit enhancement. In this group, 2 cases had cystic, solid changes with hemorrhage, and the cyst wall was mildly enhanced. The signals of cysts vary depending on the content of intracapsular proteins and the presence of bleeding. Distinguishing between cystic lesions and syringomyelia can be challenging. Syringomyelia typically exhibits clear lesion boundaries, while cystic lesions often present with blurred tumor edges.

In this study, syringomyelia was observed in Cases and . In Case , the syringomyelia was located in the conus medullaris, with mild central duct dilation observed above the lesion. As for Case , the lesions extended beyond the foramen magnum, which is likely the cause of syringomyelia in this instance. Ependymoma and hemangioblastoma often have extensive syringomyelia [20], whereas spinal PA typically has smaller syringomyelia lengths, usually smaller than the tumor length. Additionally, ependymomas of the spinal cord often show a more uniform enhancement with a clear boundary. One distinguishing feature of ependymoma from astrocytoma is the “bleeding cap sign” [21] observed on MRI, indicating changes after bleeding, which are generally absent in spinal cord PA. Ependymoma has a higher incidence of syringomyelia than spinal cord PA. Although rare, PA with spontaneous bleeding has been reported [22], with the patient developing quadriplegia within a few hours and poor functional recovery after surgery. These factors can aid in the differential diagnosis between the two conditions. Case had two separate nodules, a rare occurrence compared to previous reports. Multiple lesions formation in PA is usually due to spread along the central canal or spinal cord [23], but in some cases, it can occur in patients with neurofibromatosis [24]. In this case, enhanced MRI scans of the head and spine and clinical examination did not meet the criteria for either condition.

Extramedullary gliomas have been mentioned in studies since 1951 [25]. It was believed that these lesions originated from residual nerve cells. In previous cases, Philipson et al. mentioned a case of intradural PA at the tip of the conus medullaris, which the authors believed to originate from nerve roots [26]. Kumar et al. [14] also reported a similar case, and McBride et al. also reported multiple cases of thoracolumbar extramedullary subdural hair cell astrocytoma [27]. In this study, 2 cases were extramedullary lesions, both of which occurred in the extramedullary cauda equina region with lesions <1 cm, which is also rare. Neurogenic tumors are common in this region as well, and distinguishing them from ependymoma can be challenging due to their morphology and signal characteristics. However, ependymoma tends to have a higher probability of bleeding than neurogenic tumors, while neurogenic tumors have a higher probability of cystogenesis than spinal PA and ependymoma.

The degree of tumor enhancement is usually correlated with the degree of malignancy. The higher the degree of malignancy of the tumor, the less mature its vascular development and the greater the degree of destruction of the blood-brain barrier, resulting in local aggregation of contrast agents and further signs of enhancement [28]. PA belongs to low-grade tumors, but it can show enhancement, not because of its high malignant degree, which destroys the blood-brain barrier and leads to the accumulation of contrast agent, but because the blood vessels of PA are porous capillaries and contrast agent reaches the endothelial space of blood vessels through pores [29, 30]. In addition, there was no perifocal edema around in most PA cases, and even if some cases have mild peritumoral edema, the range of which was generally smaller than the tumor diameter, indicating its benign characteristics. PA in the spinal cord is similar to that in the intracranial lesions, with various enhancement manifestations. In previous studies [31, 19], spinal cord PA was strengthened in various ways, including nodular enhancement, slight spot-like enhancement, marginal enhancement, or even no enhancement [32, 33]. In a 2019 study, 19 nodular enhancement dominated 53.8% of cases. In our group, 58.3% of the cases were nodular enhancement, 33.3% were mild enhancement and marginal enhancement (8.3%). This is a descriptive classification with a high subjective degree, but uneven reinforcement is the main method of spinal PA reinforcement.

Spinal PA is a benign localized astrocytoma that rarely invades surrounding tissue, thus reducing the risk of malignant progression or recurrence after surgical removal. It usually shows a clear boundary between the tumor and the normal spinal cord parenchyma, making possible a 50%-81% total tumor resection rate [34, 35]. When the tumor shows cystic changes, the cystic wall must be removed along with the tumor during surgery. Although patients who had surgical resection of spinal cord PA have a 10-year survival rate of more than 90% [36], there is no clear consensus on whether the extent of tumor resection can significantly improve the overall survival rate of patients. Therefore, the scope of surgical resection of PA and the benefit of preserving nerve function still needs further exploration. The nervous system function is the best evaluation index for the postoperative functional prognosis. There is an ongoing debate about the role of chemoradiotherapy in low-grade astrocytoma, with radiotherapy being considered for cases where surgery is not feasible [37]. In this study, seven patients underwent total tumor resection, five underwent major tumor resection, and most of the patients (10 out of 12) had significant improvement in nerve function.

## 5. Conclusions

In summary, spinal PA is a low-grade astrocytoma that often presents with movement disorders followed by sensory disorders. MRI is the preferred diagnostic modality, to identify the tumor’s location, size and relationship with the spinal cord and surrounding tissues for surgical planning and prognosis monitoring. PA are most commonly eccentric and well-defined intramedullary lesions. Diffuse lesions often appear as borderless masses with cystic degeneration or bleeding, leading to early acute neurological deterioration. Extramedullary PA is rare, often characterized by small size, sometimes accompanied by cauda equina adhesion, and generally has a good prognosis. Surgery is the primary treatment for spinal cord PA, leading to a better prognosis.
